# Early control or gradual relief? Gender-specific real-world insights into omalizumab response in chronic spontaneous urticaria patients^☆^

**DOI:** 10.1016/j.waojou.2025.101163

**Published:** 2026-01-07

**Authors:** Sarah Preis, Milena Lisiecki, Tilo Biedermann, Sophia Horster, Alexander Zink

**Affiliations:** a Technical University of Munich, TUM School of Medicine and Health, Department of Dermatology and Allergy, Munich, Germany; b Institute for Medical Information Processing, Biometry, and Epidemiology, Pettenkofer School of Public Health LMU Munich, Munich, Germany; c University Hospital Munich, Department of Gastroenterology and Hepatology, Munich, Germany

**Keywords:** Vurticaria, omalizumab, gender

## Abstract

**Background:**

Chronic spontaneous urticaria (CSU) disproportionately affects women, yet gender-specific analyses in treatment response remain scarce. Real-world data on the impact of gender on omalizumab efficacy are limited.

**Objective:**

To investigate gender-specific differences in clinical characteristics, comorbidities, and response dynamics to omalizumab in a real-life CSU cohort.

**Methods:**

We conducted a retrospective, monocentric cohort study including 250 CSU patients (60% female) treated with omalizumab between 2013 and 2023. Clinical characteristics, comorbidities, laboratory parameters, and treatment timelines were analyzed. Disease control was assessed using the Urticaria Control Test (UCT) at 4 timepoints over 12 months. Statistical comparisons were performed using appropriate univariate tests and linear mixed-effects modeling.

**Results:**

Female patients had significantly higher rates of autoimmune thyroiditis, asthma, atopic eczema, allergies, and more frequently received long-term thyroid hormone therapy. While both sexes showed substantial improvement in UCT scores over time, male patients achieved faster and more stable disease control, with significantly higher UCT scores at early timepoints (p = 0.027 at timepoint 2, p = 0.004 at timepoint 3). However, overall treatment outcomes after 12 months did not differ significantly between female and male patients. Variability in response was higher among women, possibly reflecting biological heterogeneity, including hormonal status.

**Conclusion:**

Although long-term response to omalizumab is comparable between sexes, male CSU patients demonstrate faster and more consistent clinical improvement. The greater variability in female patients may be linked to immunological or hormonal cofactors. Future studies should consider menopausal status and immune profiles to identify response-modifying subgroups and support personalized treatment strategies in CSU.

## Introduction

Chronic spontaneous urticaria (CSU) is a heterogenous inflammatory skin disorder characterized by the spontaneous appearance of wheals, angioedema, or both for a duration of more than 6 weeks.^1^^,^^2^ The female-to-male ratio in CSU is estimated to be between 2:1 and 4:1, yet the biological and pathophysiological mechanisms underlying this marked sex and gender disparity remain largely unclear.^3^^,^^4^ Increasing evidence suggests that sex-based immunological and hormonal factors, such as influence of estrogen on mast cell activation or antibody production, may play a key role in disease susceptibility and immune regulation.^5^^,^^6^ In recent years, the field of gender medicine has gained significant attention, emphasizing the need to consider both sex and gender as important biological and social determinants in disease development, clinical course and therapeutic outcomes.^7^^,^^8^ CSU is particularly relevant in this context, given its female predominance, chronicity, and immune-mediated pathogenesis.^6^^,^^9^^,^^10^ Although 1 study has demonstrated clear sex-related differences in the pathogenesis of CSU, with autoimmune mechanisms being more frequent in women and idiopathic forms predominating in men, gender-specific analyses remain scarce in CSU research.^9^^,^^11^^,^^12^ This gap reflects a broader pattern across medical disciplines, where women have historically been underrepresented in clinical trials.^12^^,^^13^ As a result, therapeutic strategies are often derived from male-dominated data sets, leading to suboptimal dosing, or reduced efficacy in female patients.^12^^,^^14^ For many diseases and drug classes, women continue to experience higher rates of side effects and poorer treatment outcomes—partly due to the lack of adequately powered, sex-specific evidence.^14^^,^^15^

Findings from our systematic review ‘Is there a difference between women and men in chronic spontaneous urticaria? A systematic review on gender and sex differences in CSU patients’ support the relevance of sex and gender differences across multiple domains of CSU.^9^ We observed marked disparities in epidemiology, clinical phenotype, diagnostic indicators, comorbidities, treatment outcomes, and quality of life.^9^ Women, especially those aged 40–49 years, showed a higher disease burden and longer disease duration than men, often accompanied by greater psychosocial impact.^9^ However, when it comes to treatment outcomes, particularly with regard to biologic therapies such as omalizumab, data on sex-specific differences remain scarce and inconclusive. Only a handful of studies have addressed this question directly, and findings have been partly contradictory. While some reports suggest comparable clinical outcomes between women and men, others point to differences in remission rates, relapse frequency, or treatment resistance—often at the expense of female patients.^16^, ^17^, ^18^, ^19^, ^20^, ^21^, ^22^ In these studies, gender-specific differences were not intended to be main findings but usually emerged as incidental findings.

Taken together, these findings underline the urgent need for more robust, sex-stratified data on the efficacy and treatment dynamics of omalizumab in CSU. The aim of this study was to analyze gender-specific differences in clinical characteristics, comorbidities, and particularly in the treatment response to omalizumab in a real-world CSU cohort over a one-year follow-up.

## Methods

This study was conducted as a retrospective, monocentric cohort study with a follow-up period of 12 months. Patients with a confirmed diagnosis of CSU and an indication for the initiation of omalizumab therapy were included int the study. The indication for omalizumab treatment was based on the criteria defined in the ‘S3 Guideline on Urticaria, Part 2: Treatment of Urticaria – German language adaption of the international S3 Guideline’ by Zuberbier et al..^23^ According to this guideline, omalizumab is the approved treatment option for patients with chronic urticaria who do not show sufficient treatment response to second-generation H1-antihistamines at standard or increased doses.^23^ Omalizumab was administered according to the approved regimen for CSU, 300 mg as a subcutaneous injection every 4 weeks. Eligible patients were treated at the Department of Dermatology and Allergology, Technical University of Munich between August 1, 2013 and August 1, 2023, and were 18 years of age or older. Exclusion criteria included age under 18 years and the presence of any other severe concomitant dermatological disease. The study was approved by the Ethics Committee of the Technical University of Munich (reference number: 2024-578-S-NP).

Various patient-specific parameters were retrospectively recorded from patient files. These included the patient's age at disease onset, the time from symptom onset to diagnosis and the clinical manifestation of the disease such as wheals or angioedema at various anatomical sites. In addition, pre-existing allergological comorbidities were assessed, including food allergies, allergic rhinitis, hymenopteran venom allergy, and drug allergies (eg, to analgesics or antibiotics). Comorbidities were recorded, including asthma, atopic dermatitis, autoimmune diseases, thyroid disorders, malignancies, bacterial or viral infections, diabetes mellitus, depression, and anxiety, as well as the use of long-term medications. In addition, various laboratory parameters were collected prior to the initiation of omalizumab therapy. These included total Immunoglobulin E (IgE) levels, as well as the counts of thrombocytes, basophils, and eosinophils before treatment onset. Laboratory-based inflammatory markers such as C-reactive protein (CRP) and leukocyte counts were also recorded. Furthermore, treatment-related parameters were evaluated, including the duration of disease prior to the initiation of antihistamines and the time from disease onset to the first administration of omalizumab.

Quality of life in CSU patients was evaluated using the Chronic Urticaria Quality of Life Questionnaire (CU-Q2oL), a validated disease-specific instrument covering key domains of daily functioning and well-being.^24^^,^^25^ Disease control was evaluated with the Urticaria Control Test (UCT), a validated tool measuring patient-reported disease control over the past 4 weeks..^26^ In this study, the UCT was assessed at 4 timepoints: prior to treatment initiation, before the second dose of omalizumab, after approximately 6 months, and after 1 year of continuous therapy.

Descriptive statistics summarized the study variables, with categorial variables presenting absolute and relative frequencies and continuous variables as means ± standard deviations (mean ± SD). Group comparisons between female and male patients were conducted using Chi-square test for categorial variables (eg, comorbidities, laboratory thresholds). In cases where expected counts were low, Fishers exact test was applied. Normal distribution of the data of continuous variables was assessed using the Kolmogorov-Smirnov or the Shapiro-Wilk test. For non-normally distributed continuous variables, group comparisons between female and male patients were performed using the Mann-Whitney *U* test. Comparisons between female and male patients for continuous variables with normal distribution were performed using independent samples *t*-test.

Differences in UCT scores between female and male patients at individual timepoints were assessed using independent-samples t-tests. Comparison of timepoints were made between timepoints 1 to 4, including the UCT at baseline (timepoint 1), prior to the second dose (timepoint 2), 6 months (timepoint 3) and 12 months (timepoint 4) after the initiation of the treatment with omalizumab. To evaluate changes in disease control over time and to investigate the potential influence of gender on treatment response, a linear mixed effect model was employed. The model included timepoint as a within-subject factor, gender as a between-subject factor and a random intercept for each patient to account for intra-individual variability. A significance level of p < 0.05 was considered statistically significant.

All analyses were performed using RStudio (Version 4.2.2; R Foundation for Statistical Computing, Vienna, Austria).

## Results

Two-hundred-fifty patient files were included from the Technical University of Munich, Department of Dermatology. 150 (60%) of patients were female (median age 35 years; range 27–49 years) and 100 (40%) were male (median age 32 years; range: 25–49 years). The demographic characteristics of the patients are demonstrated in Table 1.

**Table 1:** Clinical characteristics and quality of life in female and male patients with CSU

	Female (n = 150)	Male (N = 100)	P value
Age of CSU onset (y), median (IQR)	35 [27–49]	32 [25–49]	0.443
Duration until diagnosis (months), median [IQR]	12 [2–33]	7 [3–44]	0.227
CU-Q2oL	71 ± 20	70 ± 19	0.685
Disease pattern%, (n)			
Wheals	98 (147)	97 (97)	0.456
Angioedema	70 (105)	58 (58)	0.053
Face	52 (78)	47 (47)	0.439
Mouth/nasal mucosa	12.7 (19)	10 (10)	0.312
Pharynx/Larynx with globe sensation	18 (27)	11 (11)	0.131e
Pharynx/Larynx with pronounced dyspnea	14.7 (22)	13 (13)	0.710
Trunk	3.3 (5)	2 (2)	0.418
Extremities	23.3 (35)	18 (18)	0.519
Genital area	1.3 (2)	1 (1)	0.649

Baseline data are shown in this table prior to the therapy with omalizumab.

Values are shown as median [IQR], mean ± SD, or% (n) as appropriate.

CSU, chronic spontaneous urticaria; QoL, quality of life; CU-Q2oL, Chronic Urticaria Quality of Life Questionnaire; IQR, interquartile range; SD, standard deviation.

**Fig. 1:**
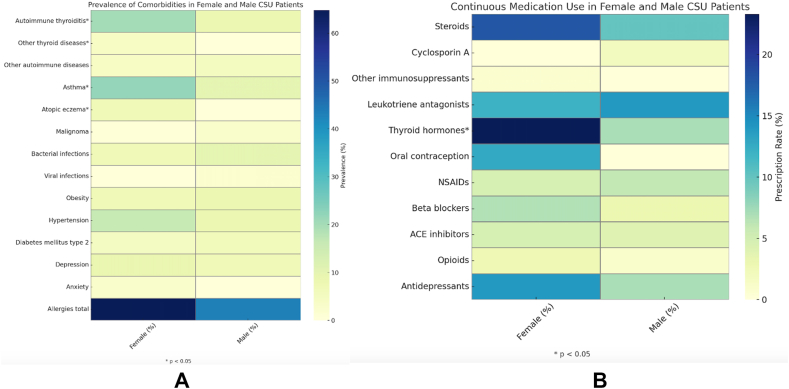
Comparison of comorbidities and long-term medication use between female and male patients, *Heatmap illustrating the prevalence (%) of selected comorbidities in female and male patients with CSU. Color intensity reflects prevalence, with darker blue indicating higher values. Statistically significant sex differences (p < 0.05) are marked with an asterisk*. *1A*: Prevalence of comorbidities in female and male patients with CSU 1B: Use of long-term medication in female and male patients with CSU.

The most common disease manifestation, wheals, affected 98% of females and 97% of males (p = 0.456), while angioedema was observed more frequently in females (70%) than males (58%, p = 0.053) (Table 1). Differences in the location of the angioedema and the affection of mucosal structures were not seen. Regarding comorbidities, females had significantly higher rates of autoimmune thyroiditis (20% vs. 8%, p = 0.007) and other thyroid diseases (p = 0.045) (Fig. 1A). This resulted in significantly more frequent continuous medication with thyroid hormones (23.3% vs. 7%, p < 0.001) (Fig. 1B). Concerning atopic diseases, females also exhibited a higher prevalence of atopic eczema (6.7% vs 0%, p = 0.005) and asthma (22% vs 10%, p = 0.010). Allergies were more common in females (64.8% vs. 43.2%) (Fig. 1A).

Other comorbidities, including malignancies (0.7% in females vs. 3% in males), bacterial infections (7% vs. 10%), and viral infections (0% vs. 2%), were observed in both groups at low and comparable rates (Fig. 1A). Similarly, obesity (6.7% in females vs. 8% in males), hypertension (16% vs. 8%), and type 2 diabetes mellitus (4.7% vs. 6%) were relatively common in both genders (Fig. 1A). Rates of depression (8.7% in females vs. 7% in males) and anxiety (2.7% vs. 0%) likewise showed no significant sex-related differences (Fig. 1A).

Comparing laboratory parameters and clinical assessments in CSU patients, a significantly higher proportion of females (60.0%, 51) had high IgE levels >100 kU/l compared to males (35.8%, 19) (p = 0.006) (Table 2). Conversely, 40.0% (34) of females and 64.2% (34) of males had IgE levels <100 kU/l. Other laboratory findings did not yield statistically significant differences between women and men. Leukocyte levels >9.0 G/l were present in the majority of females (67.7%, 88), while a higher proportion of males (65.4%, 53) had leukocyte levels <9.0 G/l (p = 0.653). Basophil levels were consistently ≤2% in both females (100%, 110) and males (100%, 69), with no gender differences observed. Eosinophil levels were predominantly <2% in females (51.8%, 59) and males (52.8%, 38). Counts between 2 and 4% were observed in 42.1% (48) of females and 37.5% (27) of males, while counts >4% were found in 6.1% (7) of females and 9.7% (7) of males. Thrombocyte levels were within the normal range (150–450 G/l) in most participants: 95.3% (123) of females and 96.2% (76) of males. A small proportion of females (3.8%, 3) and males (1.6%, 2) had levels <150 G/l, while 3.1% (4) of females—but no males—had levels >450 G/l (p = 0.235). CRP levels tended to be higher in females (39.5% > 0.5 mg/dl) than in males (28.6%), although the difference was not statistically significant (*p* > 0.05).

**Table 2:** Laboratory parameters prior to omalizumab treatment in female and male CSU patients

	Female (n = 150)	Male (n = 100)	P value
IgE value prior to omalizumab,% (n = 85/53)			
<100 kU/l	60.0 (51)	35.8 (19)	0.006
>100 kU/l	40.0 (34)	64.2 (34)	
Leukocytes prior to omalizumab,% (n = 114/94)			
< 9.0 G/l	32.1 (26)	65.4 (53)	0.653
> 9.0 G/l	67.7 (88)	31.5 (41)	
Basophils prior to omalizumab,% (n = 110/69)			
0–2%	100 (110)	100 (69)	
>2%	0 (0)	0 (0)	
Eosinophils prior to omalizumab,% (n = 114/72)			
<2%	51.8 (59)	52.8 (38)	0.609
2–4%	42.1 (48)	37.5 (27)	
> 4%	6.1 (7)	9.7 (7)	
Thrombocytes prior to omalizumab,% (n = 130/78)			
< 150 G/l	3.8 (3)	1.6 (2)	0.235
150–450 G/l	95.3 (123)	96.2 (76)	
> 450 G/l	3.1 (4)	0 (0)	
CRP prior to omalizumab,% (n = 119/77)			
< 0.5 mg/dl	60.5 (72)	71.4 (55)	0.118
> 0.5 mg/dl	39.5 (47)	28.6 (22)	

CSU, chronic spontaneous urticaria; IgE, immunoglobulin E; G/l, giga per liter; CRP, C-reactive protein.

Values are calculated based on the available number of laboratory measurements for each parameter. The number of valid observations (n = female/male) may therefore differ between parameters.

No difference was seen between the median disease duration until diagnosis of CSU between genders (12 months in females vs. 7 months in males, p = 0.227) (Table 3). Upon diagnosis, the duration of illness before the initiation of antihistamine treatment was similar between female and male patients (6 ± 31 and 2 ± 7 months, p = 0.918). When grouped by duration, 69.3% (104) of females and 69% (69) of males started antihistamine treatment within 0–5 months. A small proportion of both groups initiated antihistamine treatment after 6–11 months (2.0% females, 1% males) or after more than 12 months (4.7% females, 6% males). In 24% of both genders, the duration of illness before treatment initiation was unclear. For omalizumab initiation, the mean duration of illness was 52 ± 91 months for females and 52 ± 88 months for males, with no significant difference between genders (p = 0.658). Regarding grouped data, 44.9% (57) of females and 42.2% (35) of males initiated omalizumab treatment within 0–11 months (p = 0.904). A similar proportion of females (21.3%, 27) and males (24.1%, 20) initiated treatment within 12–35 months, while 32.3% (41) of females and 31.3% (26) of males started treatment after more than 36 months. The duration of illness was unclear for 1.6% (2) of females and 2.4% (2) of males.

**Table 3:** Time to treatment initiation with antihistamines and omalizumab in female and male patients with CSU

	Female (n = 150)	Male (N = 100)	P value
Duration of illness until initiation of antihistamine (months), mean ± SD	6 ± 31	2 ± 7	0.918
Duration of illness until initiation of antihistamine (months, grouped),% (n)			0.924
0-5	69.3 (104)	69 (69)	
6-11	2.0 (3)	1 (1)	
> 12	4.7 (7)	6 (6)	
Unclear	24 (36)	24 (24)	
Duration of illness until initiation of omalizumab (months), mean ± SD	52 ± 91	52 ± 88	0.658
Duration of illness until initiation of omalizumab (months, grouped),% (n)			
0-11	44.9 (57)	42.2 (35)	0.904
12-35	21.3 (27)	24.1 (20)	
> 36	32.3 (41)	31.3 (26)	
Unclear	6 (25)	5.9 (17)	

Values are given as mean ± SD or% (n), as appropriate.

CSU, chronic spontaneous urticaria; SD, standard deviation.

Prior to the initiation of omalizumab treatment, the mean CU-Q2oL score was 21 ± 20 for female and 70 ± 19 for male patients with no gender difference between the groups (p = 0.685) (Table 1). On the CU-Q2oL scale, higher values indicate greater impairment in quality of life; thus, both groups reported a relatively high baseline burden of disease.

**Fig. 2:**
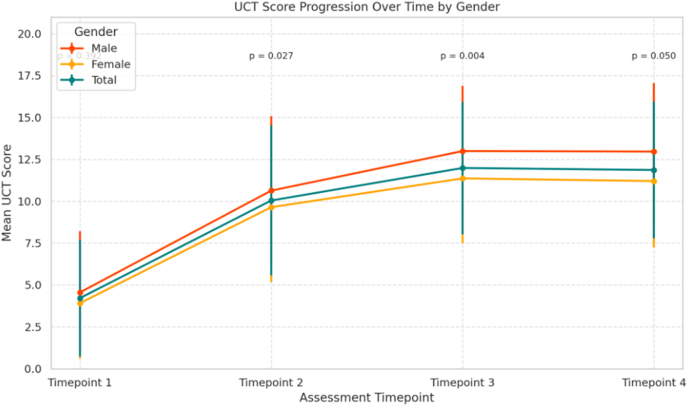
UCT score progression over time in female and male patients with CSU, *mean UCT scores and standard deviations are shown for 4 assessment timepoints. Statistically significant differences between sexes at each timepoint are indicated by corresponding* p *values*

UCT scores were assessed at 4 timepoints during the course of treatment with omalizumab and analyzed separately for male and female patients (Fig. 2). At baseline (Timepoint 1, prior to the first dose of omalizumab), mean UCT scores were 4.55 ± 3.67 in men and 3.91 ± 3.32 in women, indicating poor disease control in both groups. The coefficients of variation (CV) were 80.7% and 84.9%, respectively, reflecting high variability in initial disease burden. At timepoint 2 (prior to the second dose), UCT scores increased markedly to 10.63 ± 4.44 in men and 9.64 ± 4.47 in women. This improvement was associated with a substantial reduction in relative variability (CV 41.7% in men vs. 46.3% in women). At timepoint 3 (after 6 months after initiation of omalizumab treatment), male patients reached a mean UCT of 12.99 ± 3.90, crossing the threshold of good disease control (UCT ≥12), whereas female patients showed a mean UCT of 11.36. At timepoint 4 (after 1 year initiation of omalizumab treatment), scores remained stable with 12.96 ± 4.09 in men and 11.20 ± 3.96 in women. The coefficients of variation at both later timepoints were lower in men (30.1% and 31.5%) than in women (34.1% and 35.3%), indicating a more homogenous response in the male cohort. In the total sample, the mean UCT increased from 4.21 ± 3.48 at timepoint 1 to 11.86 ± 4.08 at timepoint 4, confirming overall treatment efficacy.

A comparison between female and male patients at each individual timepoint revealed statistically significant differences beginning at timepoint 2 (p = 0.0043), indicating that male patients achieved, on average, better, earlier and more stable control of CSU compared to female patients. To assess the influence of gender on treatment response over time, a linear mixed-effect model was applied with UCT as the dependent variable, timepoint as a within-subject factor, gender as between-subject factor and a random intercept for each patient. The model revealed a significant main effect of timepoint (all p < 0.001), confirming consistent improvement in disease control over the course of therapy. In contrast, the main effect of gender was not significant and no significant interaction was found between timepoint and gender, indicating that the pattern of UCT improvement was similar in male and female patients. Taken together, these findings suggest that both genders benefited similarly from treatment with no statistically significant differences in the trajectory of disease control as measured by UCT.

## Discussion

In this retrospective cohort study, we examined gender-specific differences in clinical characteristics, comorbidities, and treatment response in patients with CSU undergoing omalizumab therapy. While both male and female patients exhibited a high baseline disease burden and comparable time to diagnosis and treatment initiation, notable gender differences emerged in comorbid profiles and early treatment dynamics.

Female patients showed significantly higher rates of autoimmune and atopic comorbidities, particularly autoimmune thyroiditis, asthma, and atopic dermatitis. Previous population-based studies have shown that autoimmune comorbidities are more frequently observed in female CSU patients.^7^^,^^17^^,^^27^^,^^28^ A large population-based cohort study showed that CSU female patients suffer from a significantly higher incidence of atopic dermatitis and allergic rhinitis than male patients.^27^ Ghazanfar et al reported a markedly higher prevalence of autoimmune thyroid diseases in female CSU patients compared to males.^2^ Estrogens are known to modulate immune responses and may enhance humoral immunity, potentially contributing to their higher frequency of autoimmune conditions in women.^29^^,^^30^ In addition, the presence of 2 X-chromosomes, which carry numerous immune-related genes, might increase susceptibility to autoimmune dysregulation in females.^30^, ^31^, ^32^, ^33^ Women also tend to exhibit stronger T helper cell 2-type immune responses, which could explain the increased prevalence of atopic conditions such as asthma and atopic eczema.^34^ Treating the underlying autoimmune condition often leads to an improvement in CSU as well. Early identification of underlying autoimmune or atopic diseases may inform treatment strategies and improve overall disease management.^23^^,^^35^ For instance, patients with autoimmune thyroiditis may benefit from close endocrinological monitoring, while those with atopic comorbidities may require target interventions to address overlapping allergic symptoms.^36^ Early recognition of these comorbidities in CSU can support individualized treatment and ultimately improve disease outcomes.^37^ It is important to note that the comorbidity data in this study are based on self-reports, which may introduce reporting bias. In particular, men are generally less likely to seek medical attention or undergo diagnostic evaluation for non-acute or chronic conditions.^38^, ^39^, ^40^ As a result, certain comorbidities, especially those that require clinical investigations for diagnosis, such as autoimmune or allergic diseases, may be underdiagnosed or underreported in male patients. Such gender-related differences in healthcare utilization may lead to an overrepresentation of comorbidities in women, and conversely, to the underdiagnosis and possible undertreatment of male patients. What is clear, however, is the more frequent use of thyroid hormones among female patients, reflecting active treatment of their diagnosed condition.

Although laboratory investigations comparing female and male patients with CSU are limited, an early study form 2003 already reported sex-related differences.^11^ Asero et al found, that autoreactivity, assessed by the autologous serum skin test (ASST), was significantly more frequent in women (76%) than in men (35%).^11^ In the same cohort, thyroid autoantibodies were detected more often in ASST-positive patients of both sexes, and thyroid dysfunction—mainly reflected by altered TSH levels—was more prevalent among women.^11^ These results suggest that autoimmune mechanisms, including thyroid autoimmunity, may play a more prominent role in the pathogenesis of CSU in female patients. However, since ASST is not routinely performed in our clinical practice, our data cannot be directly compared with those findings. More recently, Kolkhir et al reported that eosinopenia also appears to be more common in women, further supporting potential immunologic sex differences in CSU.^41^ In our study, however, no such difference in eosinophil counts was found. Kolkhir et al further associated eosinopenia with type IIb autoimmune CSU, high disease activity and poor treatment response. In this context, the female sex also fits well as an associated factor, as it has been more frequently linked to autoimmune CSU, recurrence of symptoms, and reduced responsiveness to therapy.^42^

Encouragingly, unlike many other dermatological conditions, no gender differences were observed in the time to initiation of treatment with antihistamines or omalizumab. One of the main barriers that often delay or prevent systemic therapy in women is the lack of safety data during pregnancy or when planning to conceive.^43^ However, increasing real-world evidence now supports the safety of omalizumab as an anti-IgE antibody during pregnancy and breastfeeding.^44^^,^^45^ Therefore, it can be assumed that its use will be initiated more readily in these patient groups going forward. Regarding treatment response, both genders demonstrated a significant improvement in disease control over time, as reflected by rising UCT scores. Male patients, however, achieved better and more stable disease control earlier in the treatment course, with significantly higher UCT scores from timepoint 2 onward and lower interindividual variability. Despite these observed differences at specific timepoints, both genders benefit similarly from long-term treatment with omalizumab. The findings from our cohort, demonstrating that the long-term outcome of omalizumab treatment is independent of patient gender, are consistent with previous cohort studies investigating gender-specific treatment response to omalizumab.^19^^,^^22^ Sirufo et al prospectively followed 42 adult CSU patients treated with omalizumab, found no differences in final treatment response between sexes after the first 3 doses.^22^ In contrast, Kocatürk et al examined a CSU cohort of 110 non-responders to therapy with either cyclosporine A or omalizumab and identified female gender as a risk factor for insufficient disease control with both treatment options.^19^

In a study by Yu et al involving 59 patients with chronic urticaria, the authors investigated predictors of early treatment response, which they defined as clinical improvement within or shortly after 2 months of omalizumab therapy.^46^ Patients with an early response showed higher UCT scores and lower DLQI scores, indicating better disease control and quality of life.^46^ However, patient gender was not associated with early response. Similarly, in our cohort, we observed no gender-specific differences prior to timepoint 2, following the first administration of omalizumab. Notably, though, a short-term divergence emerged thereafter, with male patients showing a more rapid and pronounced improvement in UCT scores compared to females. In contrast to both the existing literature and our own findings, the study by Sirufo et al reported faster therapeutic response to omalizumab in women with CSU with 84.6% of female patients achieving complete remission after the first cycle, compared to only 25% of male patients (women n = 26, men n = 16).^22^ In such small patient cohorts, it can be assumed that gender is unlikely to be the most influential factor, and that the observed differences are more likely accompanied or driven by other therapy-relevant cofactors. In the context of omalizumab treatment, it is well established that cofactors such as baseline total IgE, antinuclear antibodies (ANA) and anti-thyroglobulin (anti-TG) positivity can affect the therapeutic response.^47^^,^^48^ In our cohort, the higher prevalence of autoimmune thyroid diseases and consequently likely elevated anti-TG levels in female patients may contribute to a delayed treatment response.^47^

Therefore, particular attention should be paid to the hormonal status and the associated increase in autoreactivity of female patients. As mentioned above, estrogen influences autoimmunity in women.^29^^,^^34^^,^^49^ Therefore, grouping premenopausal and postmenopausal women together when evaluating treatment must be taken into account, especially given that estrogen levels significantly decline after menopause, potentially leading to a reduction in autoimmune activity and, consequently, CSU severity.^50^ For a more nuanced analysis of treatment efficacy, it may be valuable to differentiate not only between male and female patients, but also to consider pre- and postmenopausal women. Although clear evidence regarding changes in autoimmune activity after menopause is lacking, such stratification could generate new insights and highlights the broader need for more differentiated research on postmenopausal women in clinical studies.

The lack of this distinction in current studies is also reflected in our data, particularly in the coefficient of variation (CV). Standard deviations and CVs indicated a more consistent treatment response in male patients, whereas greater variability was observed among female patients. A CV exceeding 50% is generally considered high, suggesting that patients had widely varying UCT scores at baseline, which may point to differences in disease burden and severity. The observed heterogeneity in female patients could reflect a combination of biological, clinical, and sociocultural factors, such as differences in comorbidities, autoimmune prevalence, or even symptom reporting, rather than a single explanatory variable.

## Conclusion

In summary, although male patients may achieve clinical response more rapidly or uniformly, the overall effectiveness of omalizumab appears comparable between genders. The observed variability in female patients could reflect underlying factors such as comorbidities or hormonal influences, which merit further investigation in prospective and mechanic studies. Stratification by gender or hormonal status may help to identify more homogeneous subgroups and refine therapeutic decision-making. A deeper understanding of sex-specific immune mechanisms and treatment patterns could ultimately support more personalized and effective approaches to managing CSU.

## Availability of data and materials

Due to ethical restrictions and the sensitive nature of patient data, the datasets generated and/or analyzed during the current study are not publicly available. Data may, however, be made available by the corresponding author upon reasonable request and subject to approval by the responsible ethics committee.

## Author contributions

The authors confirm contribution to the paper as follows: study conception and design: SP; data collection: ML, analysis and interpretation of results: SP, ML, AZ; draft manuscript preparation: SP; manuscript review and proof-reading: AZ, SH. All authors reviewed the results and approved the final version of the manuscript.

## Ethics statement

The study was conducted in accordance with the Declaration of Helsinki. Ethical approval was obtained from the relevant institutional review board, and a positive vote was granted. The study was conducted in accordance with the Bavarian Hospital Act (Bayerisches Krankenhausgesetz, BayKrG). According to this legal framework, the use of patient data for scientific research and publication requires neither individual informed consent nor additional approval, provided that data are analyzed retrospectively, anonymized, and handled in compliance with data protection regulations. All data used in this study were anonymized prior to analysis. The corresponding ethics committee confirmed that no further consent for publication was required.

## Authors consent for publication

All authors approved the manuscript and gave their consent for submission and publication.

## Declaration of Generative AI and AI-assisted technologies in the writing process

AI-assisted tools were used to improve readability and language.

## Funding

The study was funded by the Departement of Dermatology and Allergy, School of Medicine and Health, Technical University of Munich, Munich, Germany.

## Declaration of competing interest

SP received speaker's honoraria from Janssen, Novartis, Abbvie. ML and SH have no conflict of interest to declare. TB gave advice to or received a honorarium for talks or research grants from the following companies: ALK-Abelló, Janssen, Meda, Novartis, Phadia Thermo Fisher, Sanofi, and Celgene. AZ has been an advisor and/or received speaker's honoraria and/or received grants and/or participated in clinical trials from/of the following companies: AbbVie, ALK Abello, Almirall, Amgen, Beiersdorf Dermo Medical, Bencard Allergie, BMS, Celgene, Eli Lilly, GSK, Incyte, Janssen Cilag, Leo Pharma, Miltenyi Biotec, MSD, Novartis, Pfizer, Sanofi-Aventis, Takeda Pharma, Thermo Fisher Scientific Phadia, UCB.
